# The Responsibility of Scientists in a Time of War

**DOI:** 10.1093/function/zqac023

**Published:** 2022-05-02

**Authors:** Ole H Petersen, Alexei Verkhratsky

**Affiliations:** School of Biosciences, Sir Martin Evans Building, Cardiff University, Wales CF10 3AX, UK; Faculty of Biology, Medicine and Health, University of Manchester, Manchester M13 9PT, UK

At the time of writing this editorial, we are entering the third month of the Russo–Ukrainian war, which has brought devastations and atrocities on a scale not seen since the second world war. The consequences of the Russian invasion have reverberated across the world with people, institutions, and countries responding to the aggressor. Academia is no exception.

The Russian Empire, first under the Tsars and then under the communists, frequently exercised brutal force against its neighbours. On the night of 20^th^ August 1968, ∼200 000 Soviet troops, including soldiers from East Germany, Bulgaria, Hungary, and Poland, invaded Czechoslovakia and quickly reached Prague as there was hardly any armed resistance. The reform programme of Alexander Dubcek (“socialism with a human face”) was thereby abolished. He was forcibly taken to Moscow on 26^th^ August and made to sign the so-called 15 doctrines of the Moscow protocol. This ensured the continuation of the oppressive communist regime in Czechoslovakia that lasted for another 21 years and only came to an end with the 1989 “Velvet Revolution.” Whilst the Prague Spring was being crushed in August 1968, the 24^th^ International Congress of Physiological Sciences took place in Washington DC under the chairmanship of Wallace O Fenn. One of us (O.H.P.), although still a medical student, participated in the Congress and remembers vividly a dramatic clash between the Nobel Laureate John Eccles and the Congress Leaders. They, as well as the council of the International Union of Physiological Sciences, were adamant that the congress should be kept as a purely scientific event and that therefore no statements relating to the invasion of Czechoslovakia should be made. This was not acceptable to John Eccles, who made an unsolicited and, for the congress leadership, unwelcome intervention at the closing ceremony on 31^st^ August 1968. On that occasion, Eccles suddenly rose and launched into a very strongly worded condemnation of the Soviet Union. Several forceful attempts from the “high table” were made to stop him speaking, but Eccles was a formidable personality with a strong voice and could not be intimidated. The situation became somewhat embarrassing for the officials, as it became increasingly clear that Eccles was supported by a large number of colleagues in the audience. This clash between those preferring “business as usual” and those who feel it is a duty to condemn brutal aggression resonates to the present day, as we are now faced with a much worse and more brutal war inflicted by the Russian Federation on Ukraine.

There is obviously no controversy about the urgent need to help Ukrainian scientists fleeing the war zone and all responsible national and international scientific organisations, as well as individual scientists, have engaged and continue to be engaged in direct help programmes. However, very different views have emerged about continuing or discontinuing scientific interactions with Russian science organisations. For example, the International Science Council's (ISC) statement on the war in Ukraine^1^ is ambiguous. It expresses “deep dismay and concerns regarding the military offensives being carried out in Ukraine,” but there is no mention at all of the fact that Russian armed forces have illegally invaded Ukraine, inflicting indiscriminate atrocities on civilians. The ISC statement^1^ is at pains to emphasize that collaboration with all sides in the conflict must continue as “Ultimately the isolation and exclusion of important scientific communities is detrimental to all.” In sharp contrast, the national science academies of the G7 countries, including the world's three most important and prestigious academies—the US National Academy of Sciences, the Royal Society and the German National Academy of Sciences Leopoldina—have issued a statement^2^ that very explicitly condemns the Russian invasion of Ukraine: “The unprovoked attack against Ukraine, a democratic and independent country, is a blatant violation of international law and of core values of humanity. The Russian invasion is an assault on the fundamental principles of freedom, democracy and self-determination, which provide the basis for academic freedom and opportunities for scientific exchange and cooperation.” The statement^2^ concludes with a call to the Russian leadership “to immediately cease all military action against Ukraine and put an end to this war.” Academia Europaea (AE) has also issued a statement that is very similar to the one from the G7 academies.^3^ Furthermore, AE has given publicity to a statement from the Scientific Committee of the National Council of Ukraine on Science and Technology Development^3^ that calls for a complete severing of all scientific cooperation with Russian science and Russian scientists working in Russia. The Committee argues^3^ that “In the modern world, with information and technological know-how being the most valuable commodities, military power is largely based on the achievements of science and technology. Consequently, it is essential to isolate Russia from any such achievements.” The Ukrainian Council further states^3^ that “Not only the research funding opportunities should be withdrawn, but access to any publications, any scientific information must be ceased for the Russian academic community.” Indeed, some western governments and many international science organisations have stopped collaboration programmes with their Russian counterparts. This, in turn, has led to the Chair of the ISC and colleagues expressing their dismay, in a recent letter to Nature,^4^ at the severing of scientific ties with Russia. In their opinion, “ongoing collaboration involving Russian scientists and those from other parts of the world serves the purposes of peace and understanding.” This sentiment, however, is undermined by a shameful declaration from the Union of Russian University Rectors (Presidents).^5^ The statement, signed by 262 Russian University Presidents, starts out as follows: “In front of our eyes the events are occurring that touch upon every citizen of Russia. This is about the decision of Russia to finish, at last, the eight-year long confrontation between Ukraine and Donbass, to achieve demilitarisation and denazification of Ukraine and thus to defend ourselves from the ever-growing military threat.” Later it says: “These days, it is particularly important to support our country, our Armed Forces who ensure our safety, and to support our President who has taken perhaps the most difficult decision of his life, a very tough yet necessary decision.” In response to this belligerent and perfidious statement, the AE Board decided to suspend the membership of the only Russian University President signing the appeal, who was a member of AE.^3^

It is our personal opinion that Academia cannot ignore events such as the Russian war against Ukraine. We must respond to them and we have to find ways of contributing to the eternal struggle between the free enlightened mind and evil oppression. After all, only personal freedom makes science possible; authoritarianism crushes the free mind and thinking. We believe that the only way to achieve peace is to exert maximal pressure on opinion forming groups inside Russia and that this can only happen when these groups realize that their status in the international community is threatened. At the same time, we shall give our full support to those colleagues in Russia who have courageously opposed the war.^3^

With regard to our own subject, physiology, it is worth emphasizing that Ukraine has made substantial contributions to our current knowledge. The Ukrainian school of electrophysiology has long lasting traditions. In 1896, a young Ukrainian student, Vassily Tschagovetz, working with Julius Bernstein in Halle, Germany, applied the electrolytic theory of Walther Nernst to biological systems, which led to the hypothesis that the K^+^ selectivity of the excitable membrane is responsible for the generation of the resting membrane potential.^6,7^ Vassily Tschagovetz created the school of Ukrainian electrophysiology, holding the chair of Physiology in Kyiv from 1910 to 1941. Daniil Vorontzov, a pupil of Tschagovetz, started electrophysiological experiments in Kyiv in 1935, and in 1956 created a new laboratory of electrophysiology at the Bogomoletz Institute of Physiology in Kyiv. This institute soon became a world renowned centre for the study of the electrophysiology of nerve cells. Voronzov taught Platon Kostyuk,^8^ who went to John Eccles in Canberra in the early 1960s and upon his return to Ukraine organised the new Department of general physiology of the nervous system at the Bogomoletz Institute. In 1972, Oleg Krishtal and Vladimir Pidoplichko developed the first ever set-up for intracellular perfusion and voltage clamping of neuronal somata; a technique which played a substantial role in the subsequent development of the patch-clamp technique.^9^ In the next 20 years the electrophysiological school of Kyiv dominated physiological research in the Soviet Union. The fall of the Soviet Union and the emergence of the independent Ukraine imposed material and financial strains and precipitated the exodus of many academics. Specifically, the Kyiv school of electrophysiology was the most successful exporter with more than 70 alumni becoming professors (including one of the authors [AV] of this editorial) across the world.

Inevitably, the immediate future of science in Ukraine looks grim; a country exhausted by war cannot spare much money for fundamental research. Furthermore, as one of us wrote in a recent review:^10^ “Contrary to our intention of supporting Ukrainian scientists in Ukraine to be able to work in their own country, the poor nation, concentrating its resources on the defence against criminal Russian aggression, became a donor of highly educated scientists to the rich western world.” We owe it to this brave nation to reverse the brain drain.”
